# Dynein Light Chain Tctex-Type 1 Modulates Orexin Signaling through Its Interaction with Orexin 1 Receptor

**DOI:** 10.1371/journal.pone.0026430

**Published:** 2011-10-20

**Authors:** David Duguay, Erika Bélanger-Nelson, Valérie Mongrain, Anna Beben, Armen Khatchadourian, Nicolas Cermakian

**Affiliations:** 1 Laboratory of Molecular Chronobiology, Douglas Mental Health University Institute, Montréal, Quebec, Canada; 2 Department of Psychiatry, McGill University, Montréal, Quebec, Canada; 3 Department of Neurology and Neurosurgery, McGill University, Montréal, Quebec, Canada; Hungarian Academy of Sciences, Hungary

## Abstract

Orexins (OX-A, OX-B) are neuropeptides involved in the regulation of the sleep-wake cycle, feeding and reward, via activation of orexin receptors 1 and 2 (OX1R, OX2R). The loss of orexin peptides or functional OX2R has been shown to cause the sleep disorder, narcolepsy. Since the regulation of orexin receptors remains largely undefined, we searched for novel protein partners of the intracellular tail of orexin receptors. Using a yeast two-hybrid screening strategy in combination with co-immunoprecipitation experiments, we found interactions between OX1R and the dynein light chains Tctex-type 1 and 3 (Dynlt1, Dynlt3). These interactions were mapped to the C-terminal region of the dynein light chains and to specific residues within the last 10 amino acids of OX1R. Hence, we hypothesized that dynein light chains could regulate orexin signaling. In HEK293 cells expressing OX1R, stimulation with OX-A produced a less sustained extracellular signal-regulated kinases 1/2 (ERK1/2) activation when Dynlt1 was co-expressed, while it was prolonged under reduced Dynlt1 expression. The amount of OX1R located at the plasma membrane as well as the kinetics and extent of OX-A-induced internalization of OX1R (disappearance from membrane) were not altered by Dynlt1. However, Dynlt1 reduced the localization of OX1R in early endosomes following initial internalization. Taken together, these data suggest that Dynlt1 modulates orexin signaling by regulating OX1R, namely its intracellular localization following ligand-induced internalization.

## Introduction

OX-A and OX-B [1], also known as hypocretin 1 and 2 [2], are secreted peptide products of a common precursor, prepro-orexin. Orexins control energy homeostasis, being initially involved in the stimulation of appetite [1], as well as in the regulation of the sleep-wake cycle, where these peptides maintain and promote wakefulness [3], [4], [5], [6]. Disruption of the orexin system (loss of peptides or functional OX2R) in humans and animal models causes the sleep disorder narcolepsy [7], [8], which is characterized by excessive daytime sleepiness and cataplexy. More recently, food- and drug-associated reward and motivated behaviors have been linked to increased orexin neuron activity and thus to modulation by the orexin system [9], [10].

The implication of orexin neurons in diverse physiological functions is governed by two G-protein coupled receptors (GPCRs), OX1R and OX2R [1], [11], through activation of intracellular signaling. The cell bodies of orexin-producing neurons are exclusively found in the lateral hypothalamus but project vastly throughout the brain [12]. The distribution of orexin receptors is consistent with the projections of orexin-producing neurons. However, even though co-expression of the receptors can be observed in some brain regions, the distribution of OX1R and OX2R expression is mostly distinct and complementary [13], [14]. For example, OX1R mRNA is enriched in locus coeruleus neurons, whereas only OX2R mRNA is expressed in the tuberomammilary nucleus [14].

Orexin receptors transduce extracellular signals by activating the heterotrimeric G proteins Gq/Gi/Gs [15], [16]. Upon receptor activation, there is a robust calcium influx, which is upstream and central to orexin signaling cascades [17]. For both receptors, Gq activation signals through the phospholipase C and protein kinase C cascade to further elevate calcium and ultimately cause activation of the ERK1/2 pathway [18]. Recently, Gq-dependent phospholipase C-independent pathways were also found [19], [20]. OX1R internalization occurs in response to agonist stimulation and the receptor can be either recycled or degraded, the preferred route and its characteristics being largely undefined. Indeed, despite the various roles of orexins, little is known about the regulation of their receptors. GPCRs are extensively regulated, often through phosphorylation events and protein partners binding at their third intracellular loop and cytoplasmic carboxy-terminus [21]. Such regulatory mechanisms implicated in orexin receptor life cycle, from membrane targeting to their degradation, remain to be defined.

In the present study, we identified Dynlt1 [22], [23] as a novel partner of OX1R, and used a mutational strategy to identify the C-terminal region of Dynlt1 and the last 10 amino acids of OX1R as regions required for the interaction. OX-A-triggered ERK1/2 phosphorylation was maintained for a shorter period of time upon addition of Dynlt1 and was prolonged upon down-regulation of Dynlt1. These effects were not associated with a modulation of OX1R internalization, but rather with a change in co-localization of OX1R with an early endosome marker. Taken together, we identify Dynlt1 as a partner of OX1R that might promote the exit of OX1R from early endosomes following ligand-induced internalization, resulting in an accelerated signal termination.

## Results

### Identification of proteins interacting with orexin receptors

**Figure 1:**
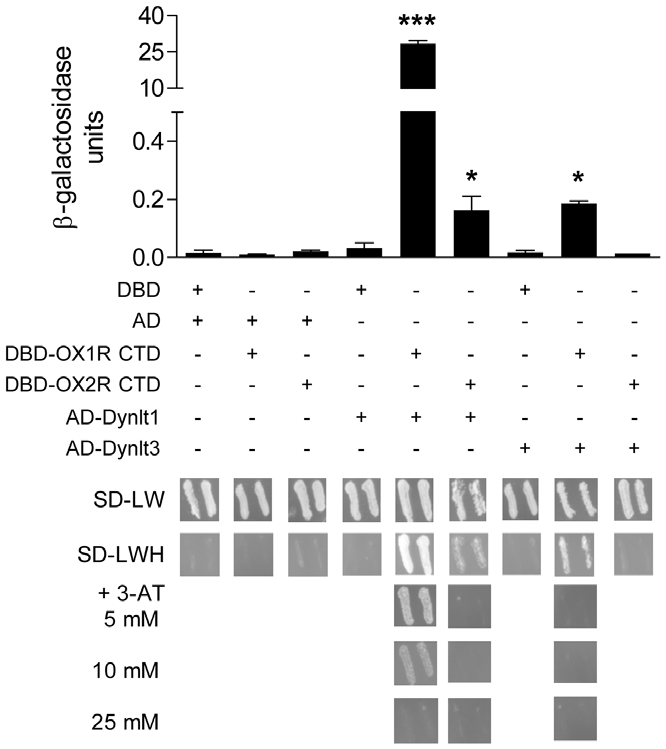
Characterization of dynein light chains Dynlt1 and Dynlt3 as partners of orexin receptors. Y187 and AH109 yeast cells were transformed with the indicated constructs and interactions were respectively assessed with ONPG liquid β-galactosidase assays (top) and HIS3 selection test (bottom). For the *HIS3* test, plates with 3-AT (3-amino-1,2,4-triazole), a competitive inhibitor of the HIS3 enzyme, were also used to assess the strength of the interaction. OX1R CTD interacted more strongly with Dynlt1 than with Dynlt3, while OX2R CTD interacted only slightly with Dynlt1. DBD, GAL4 DNA-binding domain; AD, GAL4 activating domain; OX1R CTD, carboxy-terminal domain of OX1R (a.a. 361–416); OX2R CTD, carboxy-terminal domain of OX2R (a.a. 367–460); SD–LW, medium without Leu and Trp, on which all combinations grow; SD–LWH, medium without Leu, Trp, and His, on which only yeast with interacting proteins grow. Experiments were performed 3 times, with similar results, and the average is presented. *: p<0.05 and ***: p<0.001 vs control condition (DBD/AD-Dynlt1 or DBD/AD-Dynlt3).

**Figure 2:**
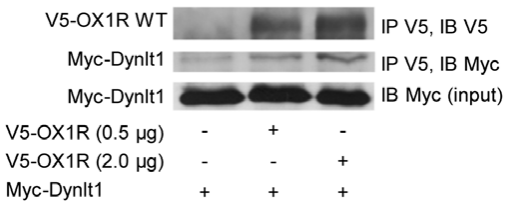
Interaction of OX1R and Dynlt1 in mammalian cells. HEK293 cells were transfected with expression vectors for Myc-Dynlt1 and V5-OX1R (0.5 or 2.0 µg DNA, full-length receptor) or corresponding empty vector. Whole-cell lysates were subjected to IP using an anti-V5 antibody and protein G-sepharose beads, followed by IB with anti-V5 or anti-Myc antibodies against V5-OX1R and Myc-Dynlt1, respectively. Equal transfection of Myc-Dynlt1 was verified by IB with anti-Myc antibody on cell extracts (input). The experiment was repeated 5 times, with similar results.

To identify proteins partners of orexin receptors, we designed a screening strategy based on the yeast two-hybrid system [24]. In order to produce a soluble protein harboring a region of GPCRs known for regulation by protein-protein interactions, we used the intracellular carboxy-terminal domain (CTD) of OX1R (amino acids L361 to S416) as bait to screen a mouse brain cDNA library. OX1R CTD was fused to GAL4 DNA binding domain (DBD) and cDNAs of the library were fused to GAL4 AD. A few hundred thousand clones were screened for histidine prototrophy and then for *β-galactosidase* expression. Sequencing of the 14 double-positive candidates identified a clone containing the full-length coding sequence of Dynlt3. This protein is one of the two members of the dynein light chain Tctex-type family, which are subunits of the dynein motor complex [23]. The function of light chains in the dynein motor is generally to bridge cargos to heavy chains, which bear the ATPase/motor activity, enabling their transport along microtubules [25]. Expanding on this initial result, we tested the interaction of OX1R CTD with the other member of the Dynlt family, Dynlt1. Physical interaction between OX2R CTD and dynein light chains was also tested in yeast. We observed that Dynlt1 interacts with the CTD of both orexin receptors, while Dynlt3 does so only with OX1R CTD (Fig. 1). However, the interaction between Dynlt1 and OX1R CTD was the strongest interaction observed, with β-galactosidase units two orders of magnitude higher than any other interaction tested. Co-immunoprecipitation (IP) experiments performed in transfected HEK293 cells using Myc-tagged Dynlt1 and V5-tagged full-length OX1R confirmed the interaction between these proteins in mammalian cells (Fig. 2).

### Characterization of domains required for orexin receptor interaction with Dynlt1 and Dynlt3

**Figure 3:**
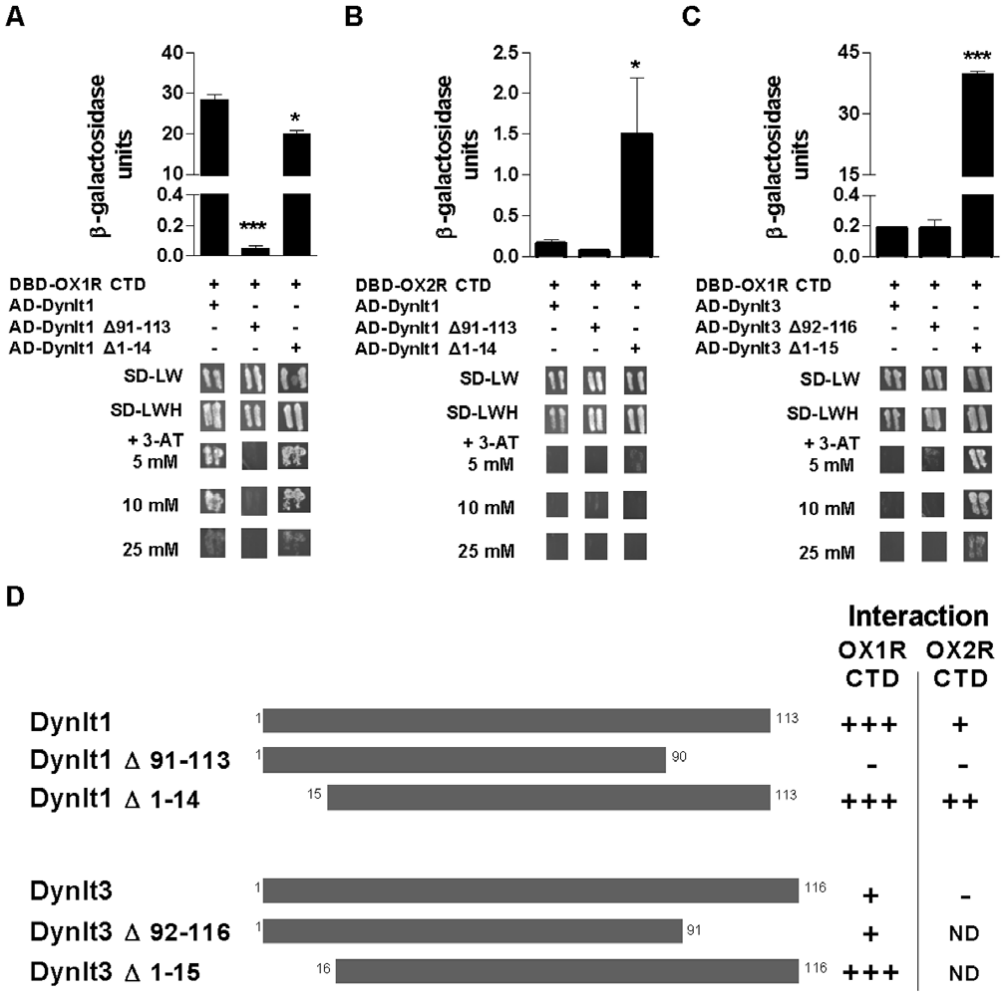
Domains of Dynlt1 and Dynlt3 involved in their interaction with orexin receptors. (**A, B**) β-galactosidase assays (top panels) and *HIS3* assays (bottom panels) were performed on yeast transformed with plasmids expressing different combinations of orexin receptor and Dynlt1/Dynlt3. The carboxy-terminal domain of Dynlt1 is required for interaction with OX1R and OX2R CTDs, while the N-terminal β sheet of Dynlt1 is dispensable for the interaction with OX1R CTD and even hinders the interaction with OX2R CTD. (**C**) The interaction of Dynlt3 with OX1R CTD remains unaltered in absence of the carboxy-terminal domain of Dynlt3, while deletion of its N-terminal β sheet favors the interaction with OX1R CTD. (**D**) Summary of Dynlt1 and Dynlt3 constructs tested and their relative interaction strength with OX1R CTD and OX2R CTD. Dynlt1 Δ91–113, Dynlt1 lacking a.a. 91–113; Dynlt1 Δ1–14, Dynlt1 lacking a.a. 1–14; Dynlt3 Δ92–116, Dynlt3 lacking a.a. 92–116; Dynlt3 Δ1–15, Dynlt3 lacking a.a. 1–15. ND, not determined. Experiments were performed 3 times, with similar results, and the average is presented. *: p<0.05 and ***: p<0.001 vs transformation with wild-type Dynlt1 or Dynlt3.

In order to delineate protein domains and amino acids involved in these novel interactions, we generated deletion mutants of Dynlt1, Dynlt3, OX1R and OX2R, and introduced point mutations in the amino acid sequence of OX1R. The carboxy-terminal region of Dynlt1 has been shown to be required for interaction with a partner [26], but structural analysis proposes a more complex model explaining how dynein light chains bridge cargo proteins to other members of the cytoplasmic dynein motor [27], [28]. Herein, using the yeast two-hybrid system, we found that OX1R CTD no longer interacts with Dynlt1 upon deletion of the carboxy-terminus of this dynein light chain (a.a. G91-I113; Fig. 3A), while this deletion non-significantly reduced the interaction of OX2R CTD with Dynlt1 (Fig. 3B). However, deletion of the carboxy-terminal region of Dynlt3 did not attenuate its interaction with OX1R (Fig. 3C). Replacement of the carboxy-terminal region of Dynlt3 (a.a. G92 to L116) by the one of Dynlt1 (a.a. G91 to I113) did not increase the ability of Dynlt3 to interact with orexin receptor CTDs (data not shown). Therefore, at least one other domain of dynein light chains confers selectivity towards cargo proteins. The amino-terminal region of Dynlt1 and Dynlt3, consisting of a protruding β sheet (amino a.a. M1 to V14 and M1 to A15, respectively) [29], also seems involved in partner selectivity or binding strength as its deletion prompted an increase of the relative strength of the interaction of dynein light chains with orexin receptor CTDs (Fig. 3B, C). This increase in interaction between OXR CTDs and Dynlt1 and Dynlt3 lacking their amino-terminal domain was observed only when interaction of OXR CTDs with WT Dynlt proteins was weak (i.e OX1R/Dynlt3 and OX2R/Dynlt1). The difference of apparent interaction strength of OX1R/OX2R with either WT or N-terminal deletion mutant Dynlt3 could be partly explained by higher expression levels of the latter (see Methods section).

**Figure 4:**
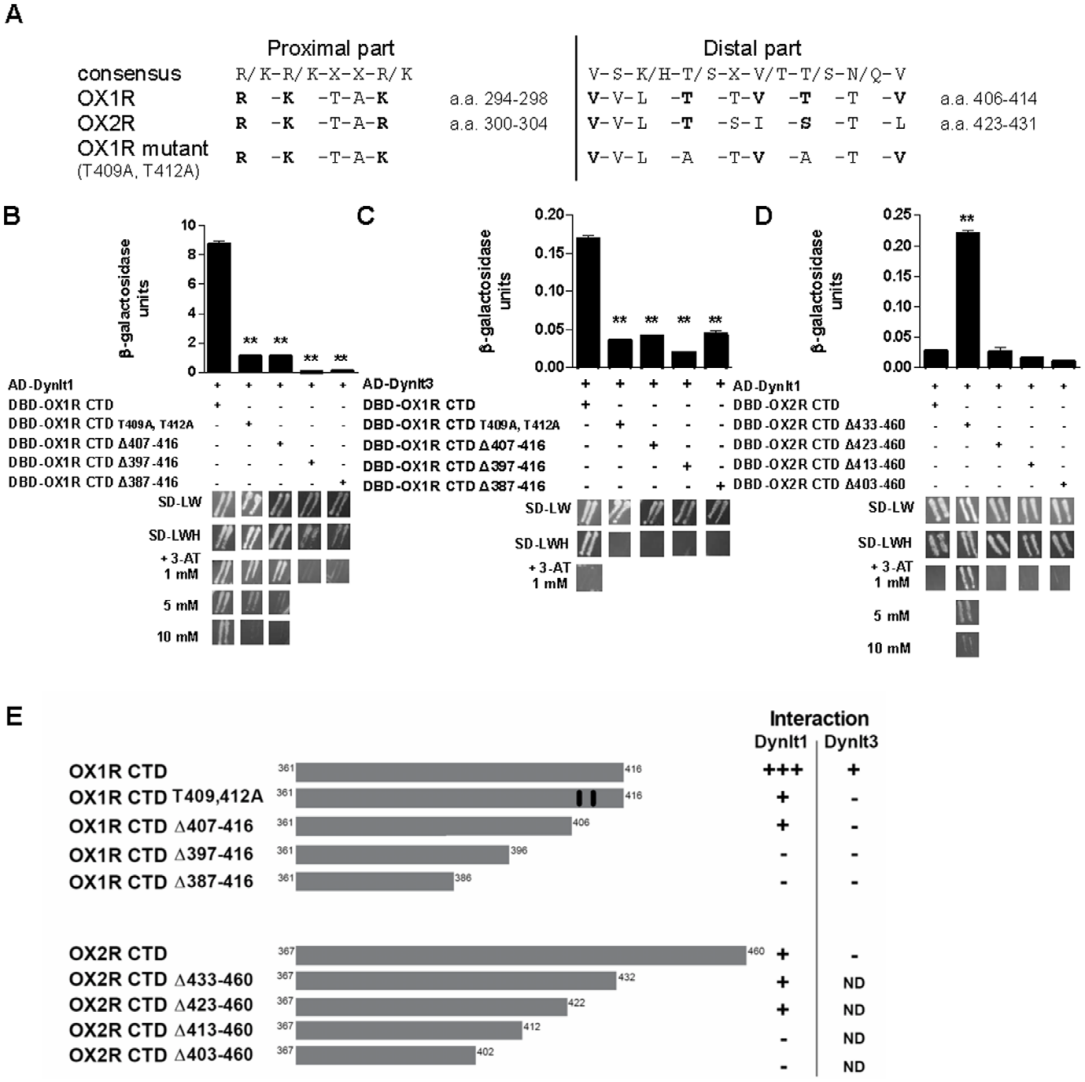
Regions of orexin receptors involved in their interaction with Dynlt1 and Dynlt3. (**A**) Identification of a putative bipartite Dynlt1-binding motif in orexin receptors. The proximal portion of the structure shown here, located in the third intracellular loop of orexin receptors, is not included in the soluble orexin receptors CTD containing the distal part and used for yeast two-hybrid assays (Fig. 1 and 3). Amino acid numbering refers to mouse sequences. Amino acids of the consensus delineated from other Dynlt1-binding proteins are shown in bold, [30], [31]. In the OX1R CTD mutant, two conserved Thr were mutated to Ala (409 and 412, numbering from full-length OX1R). (**B, C, D**) β-galactosidase (top panels) and *HIS3* (bottom panels) assays were performed on yeast transformed with plasmids expressing different combinations of orexin receptor and Dynlt1/Dynlt3. Interactions of OX1R CTD with Dynlt1 and Dynlt3 are reduced when Thr 409 and 412 of OX1R CTD are mutated into Ala. Deleting the extra amino acids of OX2R CTD favors its interaction with Dynlt1, while deleting the next 10 amino acids (comprising the distal part of the Dynlt1-binding motif) abolishes this effect. (**E**) Summary of OX1R CTD and OX2R CTD constructs tested and their relative interaction strength with Dynlt1 and Dynlt3. OX1R CTD T409, 412A, OX1R CTD with T409 and T412 mutated into alanine residues; OX1R CTD Δ407–416, OX1R CTD lacking the last 10 a.a.; OX1R CTD Δ397–416, OX1R CTD lacking the last 20 a.a.; OX1R CTD Δ387–416, OX1R CTD lacking the last 30 a.a.; OX2R CTD Δ433–460, OX2R CTD lacking the extra 28 a.a. compared to OX1R CTD; OX2R CTD Δ423–460, OX2R CTD lacking the last 38 a.a.; OX2R CTD Δ413–460, OX2R CTD lacking the last 48 a.a.; OX2R CTD Δ403–460, OX2R CTD lacking the last 58 a.a. ND, not determined. Experiments were performed 3 times and the average is presented. **: p<0.01 vs transformation with wild-type OX1R CTD or OX2R CTD.

We identified a putative bipartite Dynlt1-binding motif [30], [31] located in the third intracellular loop of full-length orexin receptors and in the last 10 amino acids of the CTD (Fig. 4A). Mutating two threonine residues of the distal part of the motif (T409A and T412A) significantly reduced the interaction of OX1R CTD with Dynlt1 and Dynlt3 (Fig. 4B,C). Successive deletions of 10 amino acids starting from the carboxy-terminus of OX1R indeed revealed that most of its interaction with Dynlt1 and Dynlt3 is mediated by the last 10 residues (Fig. 4B,C). Residual physical contact between OX1R CTD and Dynlt1 was abolished when the last 20 amino acids of OX1R CTD were deleted (Fig. 4B). The selectivity of Dynlt1 towards OX1R versus OX2R lies, at least in part, in the extra residues of OX2R forming a longer carboxy-terminal tail because deleting a.a. A433 to V460 of OX2R led to an increase of its interaction with Dynlt1 (Fig. 4D). A summary of the domains involved in the interaction between OXR CTD and Dynlt1/3 is provided in Fig. 3D and Fig. 4E.

**Figure 5:**
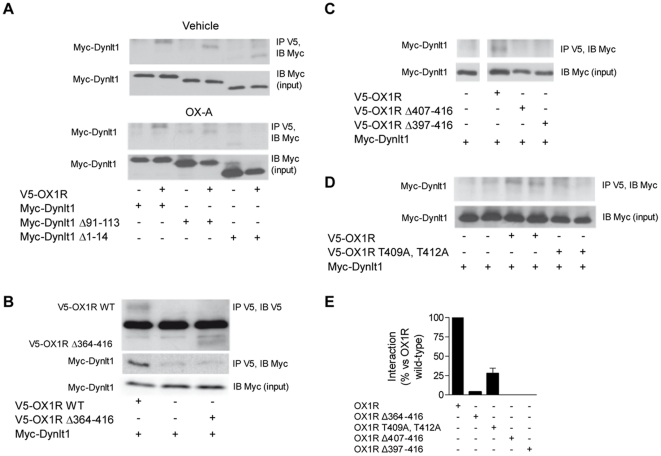
The effect of Dynlt1 mutations and OX1R mutations on their interaction in mammalian cells. (**A**) HEK293 cells were transfected with the indicated constructs and stimulated with orexin-A (OX-A) or vehicle (water) for 30 min. Whole-cell lysates were then subjected to IP as described in Fig. 2. OX1R interaction with Dynlt1 is not disrupted by the presence of deletion mutants of Dynlt1 after addition of vehicle, but it is reduced (compared to wild-type Dynlt1) after stimulation with OX-A. (**B**) HEK293 cells were transfected with the indicated constructs and whole-cell lysates were subjected to IP. Removing the CTD of OX1R abolishes the interaction of OX1R with Dynlt1. (**C, D**) Co-IP experiment performed on whole-cell lysates obtained from HEK293 cell transfected with the indicated constructs (Myc-Dynlt1 and V5-OX1R deletion mutants). Removing the last 10 amino acids from OX1R abolishes its interaction with Dynlt1, while mutating two threonine residues for alanine in the putative Dynlt1-binding motif (Fig. 4A) blunts the amount of Myc-Dynlt1 co-immunoprecipitated with V5-OX1R. (**E**) Quantification of Western blotting bands obtained in panels B, C and D. All transfection conditions and co-IP experiments were performed at least twice.

Selected deletions and mutations were then introduced in Myc-tagged Dynlt1 and full-length V5-tagged OX1R, and the constructs were tested in co-IP experiments in HEK293 cells. We found that in resting conditions, the interaction of OX1R with Dynlt1 persisted even when the carboxy-terminus or the amino-terminus of Dynlt1 is absent. However, stimulation of transfected cells with OX-A highlighted the importance of both Dynlt1's domains for the sustained interaction with OX1R (Fig. 5A). With this mammalian system, we also confirmed that the CTD of OX1R (Fig. 5B), its last 10 and 20 amino acids (Fig. 5C) and more specifically residues T409 and T412 are crucial for a maximal interaction with Dynlt1 (Fig. 5B–D).

### Modulation of OX-A-activated ERK proteins by Dynlt1

**Figure 6:**
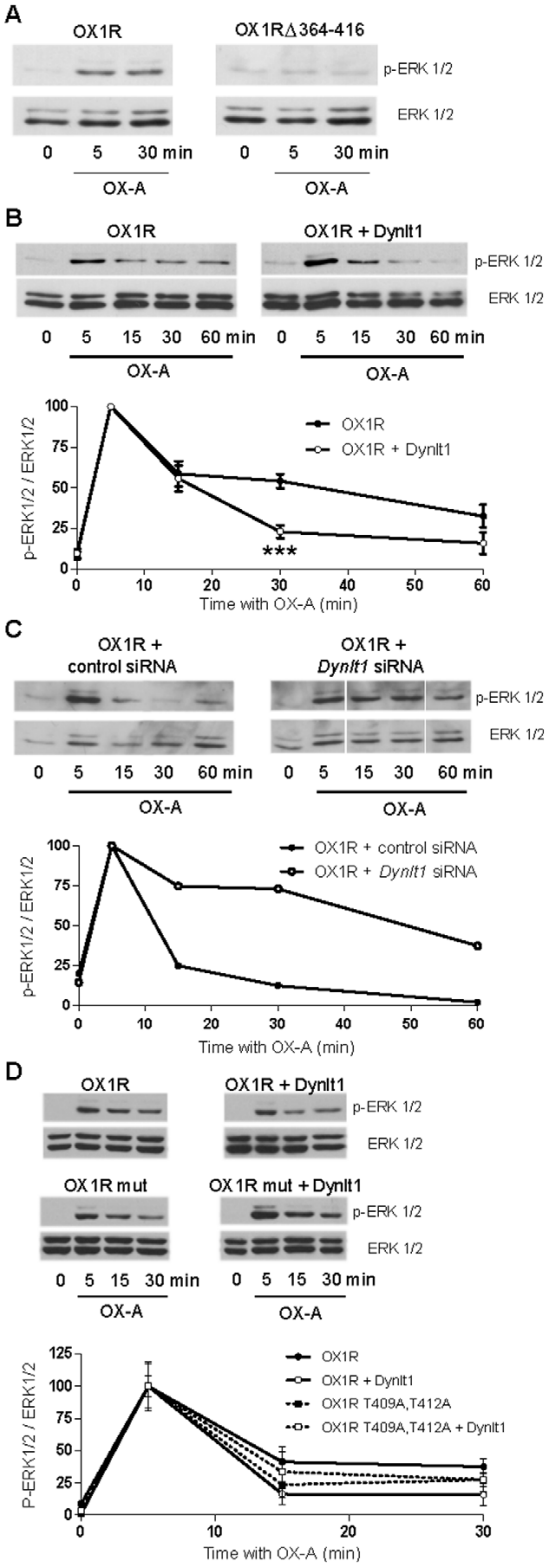
Modulation of OX1R-mediated signaling by Dynlt1. (**A**) HEK293 cells were transfected with a V5-OX1R or V5-OX1R Δ364–416 expression vector and stimulated with 500 nM OX-A for the indicated times. Protein extracts were analyzed by SDS-PAGE and Western blotting with anti-phospho-ERK1/2 and anti-ERK1/2 antibodies. ERK1/2 are quickly phosphorylated after OX-A stimulation and OX1R CTD is essential to this response. V5-OX1R Δ364–416, V5-OX1R lacking its CTD. (**B–D**) Transfected HEK293 cells were stimulated with 100 nM OX-A for the indicated times and processed as in (A). (**B**) Co-expression of Dynlt1 leads to a less sustained ERK1/2 activation in response to OX-A. Blots are from a representative experiment. The graph shows a combination of 4 independent experiments. ***: p<0.001 vs data without Dynlt1 transfected (ANOVA followed by post-hoc analysis at the different times). (**C**) Down-regulation of Dynlt1 by a siRNA (10 nM, expression reduced by 88%) leads to a sustained activation of the ERK1/2 pathway in response to OX-A. This experiment was repeated twice, each in duplicates, with similar results. The blots and the graph present the results from one of these experiments. (**D**) Dynlt1 does not lead to a less sustained ERK1/2 activation in response to OX-A when co-expressed with OX1R mutant T409A, T412A. This experiment was repeated twice, each in triplicates, with similar results. The blots and the graph are from one of these experiments.

We then verified the biological significance of the OX1R/Dynlt1 interaction. To do so, we used the activation of ERK1/2 by phosphorylation after cell stimulation with OX-A as a read-out of OX1R function. OX-A did not elicit phosphorylation of ERK1/2 in HEK293 cells that were not transfected with pSG5-V5-His-OX1R (data not shown). However, ERK1/2 was transiently phosphorylated after stimulating HEK293 cells expressing V5-tagged OX1R with OX-A, reaching a maximal activation at 5 min, and declining within 60 min (Fig. 6A,B). This signaling event required the presence of OX1R CTD (Fig. 6A). In this system, over-expression of Dynlt1 did not modify the levels of ERK1/2 phosphorylation reached at 5 min, but led to less sustained response of ERK1/2 phosphorylation (Fig. 6B). This indicates that Dynlt1 can modulate OX1R responses elicited by OX-A. As an additional support, the down-regulation of Dynlt1 using a siRNA approach in HEK293 cells permitted a prolonged activation of ERK1/2 without altering the maximal level of phosphorylation observed after 5 min (Fig. 6C). Over-expression of Dynlt1 did not modify the kinetics of ERK1/2 phosphorylation in response to OX-A when co-transfected with the OX1R T409A, T412A mutant (Fig. 6D). Since this OX1R mutant showed blunted interaction with Dynlt1 (Fig. 4C, 5D), we infer that the effect of Dynlt1 on the response to OX-A is specific to orexin receptor signaling as it requires the intact interaction between both proteins.

### Regulation of OX1R intracellular localization by Dynlt1

Since Dynlt1 is a component of the cytoplasmic dynein motor and can alter responses elicited by OX-A, we studied the regulation of OX1R cellular localization by Dynlt1. We first measured the amount of OX1R located at the cytoplasmic membrane under resting conditions using a cell surface ELISA. A V5 tag added at the extracellular amino-terminus of OX1R allowed for the specific detection of membrane-bound OX1R in absence of permeabilization. Over-expression or down-regulation of Dynlt1 did not significantly change the level of membrane-bound OX1R (Table 1). In the latter experiment, presence of Dynlt3 could have compensated for the absence of Dynlt1, as both dynein light chains were found to interact with OX1R. However, no significant alteration of OX1R level at the membrane was detected upon application of siRNAs against both Dynlt1 and Dynlt3 (Table 1).

**Table 1:** Levels of OX1R in resting conditions at the plasma membrane in presence or absence of Dynlt1.

	Membrane-bound V5-OX1R
	(% of control)
Over-expression of Dynlt1	98.5±5.3 (control = 100±7.9)
Down-regulation of Dynlt1	88.3±14.2 (control = 100±9.1)
Down-regulation of Dynlt1 and Dynlt3	109.7±19.7 (control = 100±8.2)

Control refers to a different transfection for each experiment. Empty pCS2, 10 nM or 20 nM negative control siRNA were used respectively as control for the transfection of pCS2-Dynlt1, *Dynlt1* siRNA or a mix of *Dynlt1* and *Dynlt3* siRNAs.

**Figure 7:**
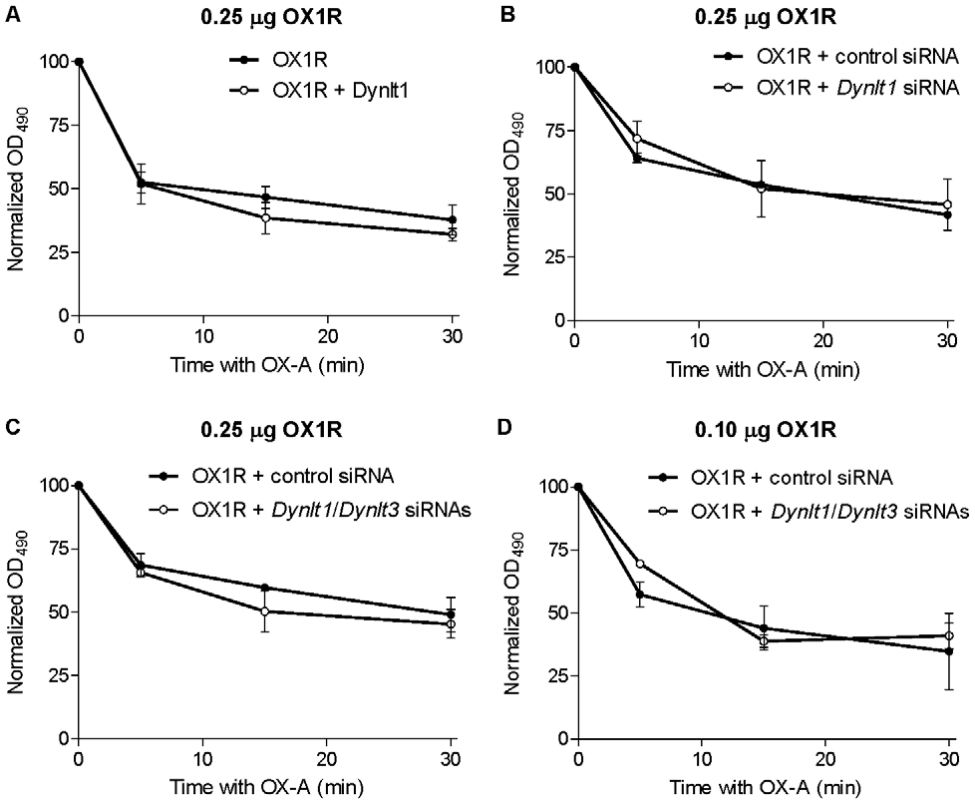
Agonist-induced internalization of OX1R in presence or down-regulation of Dynlt1/Dynlt3. Cell surface detection of OX1R was measured by ELISA. HEK293 cells were transfected with either 0.1 (A) or 0.25 µg (B, C, D) control pSG5-V5-His or pSG5-V5-His-OX1R, in combination with: (**A**) pCS2-Myc or pCS2-Myc-Dynlt1, (**B**) 10 nM of either control siRNA or *Dynlt1* siRNA (80% down-regulation), or (**C, D**) either 20 nM control siRNA or a combination of *Dynlt1* and *Dynlt3* siRNAs at 10 nM each (75% and 80% down-regulation, respectively). Cells were then treated with OX-A for up to 30 min. Normalized OD values refer to OD values that have been corrected (background-subtracted) and then expressed relative to basal conditions (% of value before stimulation for each condition). For all panels, there is maximum OX1R at the membrane at basal conditions (0 min; without OX-A) and the receptor is internalized (represented by loss at the membrane) after OX-A treatment. Results represent the mean ± SEM of 3 experiments, each performed in triplicate (except for panel D; results represent the mean ± SEM of 2 experiments). There is no statistical significance for the effect of over-expressing or down-regulating Dynlt1 and/or Dynlt3 as assessed by two-way ANOVA.

These results, indicating that Dynlt1 does not regulate OX1R membrane targeting, are in accordance with the generally minus-end directed cargo transport [32] governed by the cytoplasmic dynein motor [33]. Since the internalization of GPCRs commonly involves minus-end directed transport of vesicles along the endocytic pathway [34], we monitored the internalization of OX1R after stimulating V5-OX1R-transfected HEK293 cells with OX-A. The cell surface ELISA showed that over-expression of Dynlt1 (Fig. 7A), down-regulation of Dynlt1 (Fig. 7B), or down-regulation of both Dynlt1 and Dynlt3 (Fig. 7C) did not modify the kinetics of OX1R disappearance from the plasma membrane. Down-regulation of Dynlt1 and Dynlt3 in presence of lower amounts of receptor was also without effects (Fig. 7D). It remains possible that the remaining protein expressed from the residual RNA be sufficient to perform the action on OX1R. However, these data, together with alteration of OXR signaling (P-ERK) at similar levels of down-regulation of *Dynlt1* RNA, indicate that Dynlts do not regulate the initial internalization of OX1R after stimulation with OX-A.

**Figure 8:**
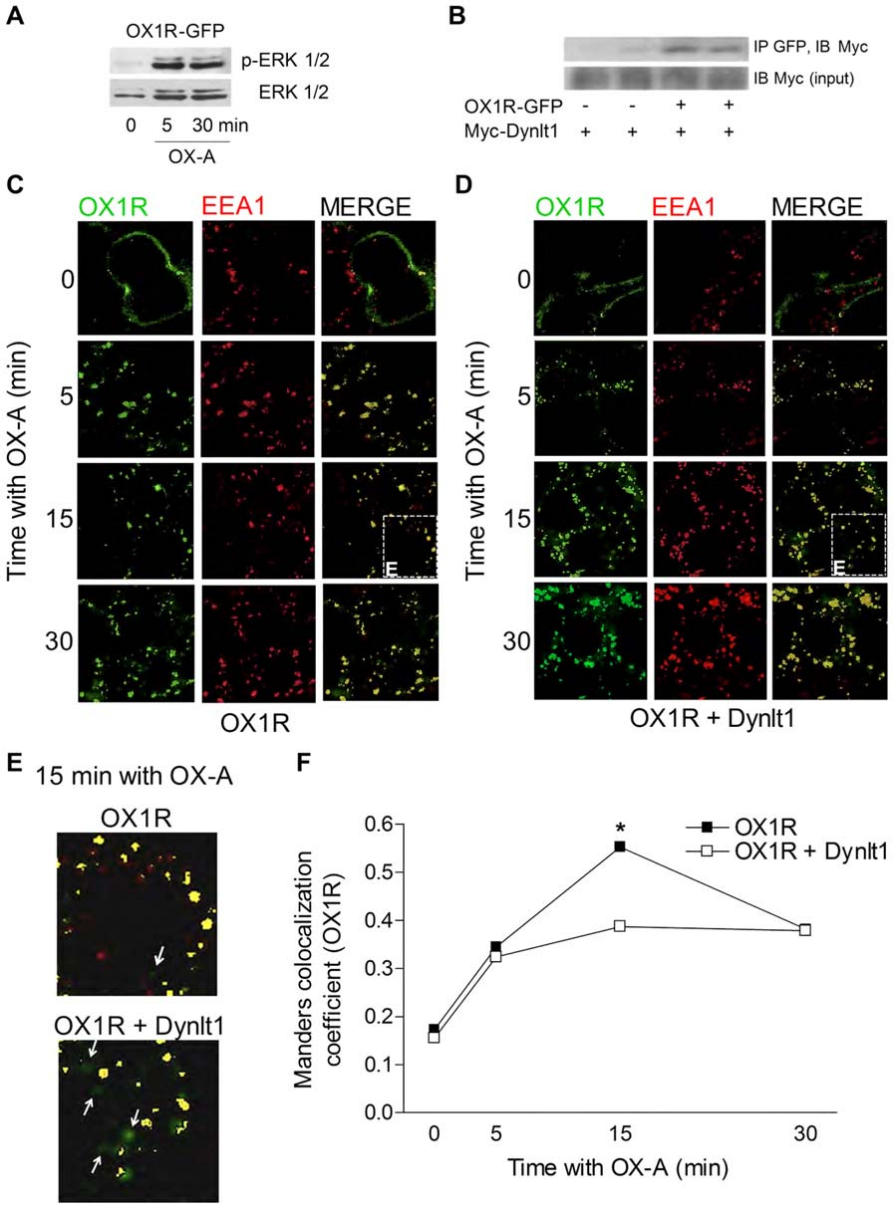
Dynlt1 alters the intracellular localization of OX1R following ligand-induced internalization. (**A**) HEK293 cells were transfected with pEGFP-N1-OX1R and stimulated with OX-A for the indicated times. IB analysis of whole cell lysates shows that ERK1/2 phosphorylation can be achieved after stimulation of this OX1R-GFP fusion protein with OX-A. (**B**) HEK293 cells were transfected with pCS2-Myc-Dynlt1 and pEGFP-N1-OX1R or corresponding empty vector. Whole-cell lysates were subjected to IP with an anti-GFP antibody to immunoprecipitate OX1R-GFP and anti-Myc antibody to reveal Myc-Dynlt1. Equal transfection of Myc-Dynlt1 was verified by IB with anti-Myc antibody on cell extracts (input). (**C, D**) HEK293 cells stably expressing OX1R-GFP were transfected with control plasmid (**C**) or pCS2-FLAG-Dynlt1 (**D**), stimulated with OX-A for 5–30 min or left un-stimulated (0 min) and analyzed by fluorescent microscopy. Cells were labeled with an antibody specific for a marker of early endosomes, EEA1. Maximum co-localization of green pixels with red pixels occurs after 15 min OX-A in control conditions. However, when over-expressing Dynlt1, co-localization is rather similar across OX-A stimulation times. Left panels: green GFP fluorescence; OX1R, middle panels: EEA1 labeling, red ALEXA 568 secondary antibody; early endosomes, and right panels: the merged images. (**E**) Magnification of merged fields from panels C and D (white squares) after 15 min OX-A without or with Dynlt1 over-expression. White arrows indicate OX1R-GFP signal (green) not co-localized with endosome marker (red). (**F**) Manders' co-localization coefficient for green channel (OX1R-GFP) was generated on images that were co-localized using Openlab software. Manders' co-localization coefficient (OX1R): co-localized green and red pixels divided by total number of green pixels over threshold. Results represent the mean ± SEM of triplicates from one experiment, where at least 5 fields with 1–3 cells in each were analyzed for each time point. This experiment was done twice with similar results (three times for the 0 and 15 min time points). The co-localization of OX1R-GFP with the EEA1 marker for endosomes, after 15 min OX-A, is decreased upon Dynlt1 over-expression. *: p<0.05 when compared to control transfection (without Dynlt1) after 15 min OX-A.

Lastly, we studied the fate of OX1R upon its internalization under basal levels or after over-expression of Dynlt1 using fluorescence microscopy. HEK293 cells stably transfected with green fluorescent protein (GFP)-tagged OX1R were generated and verified for OX1R functionality. Adding GFP at the amino-terminus of OX1R led to the production of a non-functional fusion protein, as indicated by its intracellular localization in resting conditions and by the absence of ERK1/2 phosphorylation upon exposition to OX-A (data not shown). However, the carboxy-terminal tagged OX1R-GFP was located at the plasma membrane in resting conditions, and was internalized and led to phosphorylation of ERK1/2 after stimulation with OX-A (Fig. 8A,C). Importantly, it retained the interaction with Dynlt1 (Fig. 8B). Using this construct, we found increased co-localization of the OX1R-GFP signal (green) with the early endosome marker EE1A (red) following stimulation with OX-A. The co-localization of OX1R and early endosome (yellow color) peaked at 15 min after stimulation with OX-A (Fig. 8C,E). However, over-expression of Dynlt1 caused a significant reduction of this maximal co-localization (more green fluorescence seen in the merged picture; see Fig. 8D,E,F), suggesting that the presence of Dynlt1 in the cells leads to a reduction of the time spent by OX1R in early endosomes (Fig. 8D,E).

## Discussion

In this study, we identified dynein light chains Dynlt1 and Dynlt3 as novel partners of orexin receptors. A consensus Dynlt1-binding motif in the CTD of OX1R is required for the interaction to occur. Although it is not involved in membrane targeting of OX1R or its internalization per se, Dynlt1 appears to regulate the transition of OX1R in early endosomes. This, coupled to an early termination of OX-A-induced ERK1/2 activation in presence of Dynlt1, reveals that this member of the dynein complex is a potential modulator of orexin biological functions.

### OX1R: a novel cargo of dynein

The list of cargos (proteins associating with a motor complex) for Dynlt1 and Dynlt3, which are members of the Tctex1/Dynlt family of dynein light chains, has expanded in recent years [35], [36], [37], [38], [39]. The function of proteins from various families is governed by cytoplasmic or axonemal dynein motor complexes. Among the proteins previously shown to interact with Dynlt1 are two other GPCRs, parathyroid hormone receptor (PTHR) and Rhodopsin [31], [40]. Cargos have also been described for other families of dynein light chains (LC8/Dynll and Roadblock/Dynlrb). Proteins bound to a homodimer of light chains are bridged to dynein heavy chains, which bear the ATPase activity, by light-intermediate chains and intermediate chains [27]. In accordance with cytoplasmic dynein's minus-end directed transport [41], Dynlt1 is required for internalization of many known cargos. For example, it has been suggested that Dynlt1 is involved in the internalization of PTHR following agonist stimulation [31]. The reverse is observed with Rhodopsin, as Dynlt1 governs its delivery to the apical membrane [40]. Herein, we found that Dynlt1 modulates the subcellular localization of OX1R after agonist-dependent internalization, rather than its targeting to the membrane.

Both PTHR and Rhodopsin interact specifically with Dynlt1. In the present study, we found that OX1R interacts more strongly with Dynlt1 than with Dynlt3 in yeast, and only the OX1R and Dynlt1 interaction was confirmed in mammalian cells. This is consistent with the fact that much of the amino acids that differ between Dynlt1 and Dynlt3 are located on structure surfaces forming the putative cargo binding domain [28]. While we report a bipartite consensus Dynlt1-binding motif in OX1R (Fig. 4A), it is difficult to define a Dynlt3-binding motif because there are not enough known partners of Dynlt3.

In the brain, where orexin receptors are mostly studied, the expression pattern of Dynlt1 and Dynlt3 appears to be non-overlapping in some regions such as the hippocampus and the cerebral cortex [42]. Therefore, it is possible that different members of the dynein complex are involved in a similar regulation of OX1R, but doing so in specific regions. This would be consistent with the fact that orexin receptors located in distinct brain regions are linked to specific biological functions [5], [9]. Moreover, the roles of Dynlt1 and Dynlt3 in the dynein complex are most likely not redundant but rather competitive, since over-expression of Dynlt3 has been shown to displace endogenous Dynlt1 bound to the dynein complex and to disrupt Dynlt1-related functions [31], [43].

### Dynlt1: a novel regulator of orexin signaling

GPCRs interacting with Dynlt1 present one or both parts of the bipartite binding motif. The distal motif found in Rhodopsin CTD is required to ensure regulation by dynein [40], while either or both the proximal (third intracellular loop) and distal (CTD) motifs in PTHR may be important for receptor localization and internalization [31]. For some GPCRs, the third intracellular loop is highly regulated by phosphorylation and protein partner binding [44]. Structural studies defining the stoichiometry of the interaction would be required to show that one Dynlt1 protein binds to both motifs, or that two Dynlt1 proteins are required to occupy the OX1R binding sites. OX1R CTD intuitively lacks the proximal Dynlt1-binding motif identified in the third intracellular loop of the full length GPCR (R294-K298). This portion of the binding motif may be required to achieve an interaction between OX1R and Dynlt1 in mammalian cells, but not in a different system such as yeast nuclei in which we detected the interaction between OX1R CTD and Dynlt1. Alternatively, another molecule could serve as an adapter between OX1R and Dynlt1 in mammalian cells. The requirement of such an adapter protein seems unlikely since OX1R displays a Dynlt1-binding site and, as mentioned, we detected the interaction in yeast two-hybrid assays. However, a post-translational modification could be needed to promote the interaction between OX1R and Dynlt1. For instance, the recruitment of the protein tyrosine phosphatase SHP-2 to OX1R CTD was shown to rely on OX-A-dependent human OX1R Y83 [19] and Y358 phosphorylation [20]. Interestingly, the regulation of orexin function by Dynlt1 may be restricted to OX1R-mediated signaling. Accordingly, even though OX2R bears the consensus Dynlt1-binding motif, the presence of extra carboxy-terminal amino acids compared to OX1R was found to prevent the interaction with Dynlt1 (Fig. 4D).

We found that OX-A-induced ERK1/2 activation terminates faster when Dynlt1 is over-expressed (Fig. 6B). This result could involve the modulation of alternative signaling pathways by Dynlt1. As such, G-protein-independent signaling, including ERK1/2 phosphorylation, can occur through β-arrestin-2 [44] and is proportional to the amount of time spent in endosomes by the GPCR/β-arrestin-2 complex after ligand-dependent receptor internalization via clathrin-coated vesicles [45], [46]. Residues of OX1R CTD required for association with β-arrestin-2 are also involved in ERK1/2 activation [47], [48]. We identified that the two threonine residues of OX1R involved in these responses are also important for the interaction of the receptor with Dynlt1. Additionally, we found that blunting OX1R interaction with Dynlt1 by mutating these two sites (T409A, T412A) prevents the effect of Dynlt1 on ERK1/2 responses elicited by OX-A. Taken together with our data showing an earlier decrease of OX1R localized in early endosomes in cells over-expressing Dynlt1 and stimulated with OX-A, it is possible that Dynlt1 competes with β-arrestin-2 for binding to OX1R. This would lead to less sustained β-arrestin-2-dependent responses, as observed with ERK activation. Moreover, Dynlt1 may be implicated in another regulatory mode of GPCRs and G proteins involving Gβγ subunits [49]. Indeed, Dynlt1 was independently identified as AGS2, an activator of G protein signaling [50]. Dynlt1 regulates neurite outgrow through its direct interaction with Gβγ [51] and we thus cannot exclude the possibility that it modulates orexin signaling by acting as an effector of Gβγ functions.

Internalization of OX1R was not altered when Dynlt1 was over-expressed or down-regulated (Fig. 7). Since clathrin-coated vesicles fuse with early endosomes [45], we infer that the amount and kinetics of internalized OX1R getting to early endosomes were not modulated by Dynlt1. Consequently, the exit from early endosomes would be the step modulated by Dynlt1. Additionally, Dynlt1 could also regulate the intracellular location of OX1R beyond the early endosome compartment, including recycling endosomes and lysosomes.

### Conclusion

Orexin receptors are targeted by novel pharmacotherapies [52]; the importance of characterizing events downstream of their activation is crucial. Herein, we revealed that Dynlt1, a subunit of the dynein motor complex, interacts with OX1R in mammalian cells. It appears to act as a regulator of OX1R intracellular localization and signalization, reducing the time spent in early endosomes and promoting signal termination. The modulation of the expression of Dynlt1, or modifying its association with orexin receptors, could thus become a new strategy to control orexin-dependent biological functions.

## Materials and Methods

### Plasmids and cloning

#### OX1R and OX2R constructs

We amplified mouse OX1R (Accession No.: NM_198959, GenBank) CTD (amino acids 361 to 416) and mouse OX2R (Accession No.: NM_198962, GenBank) CTD (amino acids 367 to 460) by PCR (using mouse brain cDNA). The PCR products were inserted between EcoRI and SalI sites in-frame with the DBD of the yeast transcription factor GAL4, in pGBKT7 plasmid (Clontech, Mountain View, CA). Deletion mutants of OX1R CTD (Δ407–416, Δ397–416 and Δ387–416, lacking the last 10, 20 and 30 amino acids, respectively) were created by inserting a stop codon at the appropriate position. Similarly, deletion mutants of OX2R CTD (Δ433–460, Δ423–460, Δ413–460 and Δ403–460, lacking the extra amino acids 433 to 460 compared to OX1R, and subsequent deletion of 10, 20 and 30 residues, respectively) were created. Point mutations in OX1R (T409A, T412A) were introduced using PCR site-directed mutagenesis. The various OX1R CTD fragments were then inserted between the EcoRI and SalI sites of pGBKT7, in frame with the sequence encoding GAL4 DBD.

Full-length OX1R construct was created by PCR amplification, using oligonucleotides targeted to the coding sequence of the mouse OX1R (NM_198959) and inserted between EcoRI and XhoI sites of pSG5-V5-His plasmid [a SV40 promoter plasmid from Stratagene (Cedar Creek, TX, USA), which has been engineered to contain V5 and His_6_ tag sequences]. The various mutants of full-length OX1R were produced as described above. Enhanced GFP-OX1R fusion protein was prepared by subcloning OX1R from the pSG5-V5-His-OX1R construct into pEGFP-C1 plasmid (Clontech). Full-length OX1R-EGFP fusion protein was prepared by PCR amplification as above, but using a reverse oligonucleotide lacking the STOP codon and designed to create a protein in frame with the downstream EGFP coding sequence. The EcoRI/XhoI-digested insert was cloned in the pEGFP-N1 plasmid (Clontech).

#### Dynlt1 and Dynlt3 constructs

The coding sequences for mouse Dynlt1 (Accession No.: NM_009342, GenBank) and Dynlt3 (Accession No.: NM_025975, GenBank) were amplified by PCR (using mouse brain cDNA). Carboxy-terminal deletion mutants (Dynlt1 Δ91–113 and Dynlt3 Δ92–116) were created using PCR site-directed mutagenesis, by inserting a stop codon at the appropriate position. Amino-terminal deletion mutants (Dynlt1 Δ1–14 and Dynlt3 Δ1–15) were created by PCR amplification with forward oligonucleotides lacking the first 42 or 45 bases of the coding sequence, respectively. The various Dynlt1 and Dynlt3 fragments were then inserted between the EcoRI and XhoI sites of pGADT7 (Clontech), in frame with the GAL4 activation domain (AD).

The pCS2-Myc-Dynlt1 construct (a CMV promoter plasmid with 5 tandem Myc tag sequences) was created by subcloning from pGADT7-Dynlt1 (wild-type or mutant), using EcoRI and XhoI. Subsequently, pCS2-FLAG-Dynlt1 was prepared from pCS2-Myc-Dynlt1. A FLAG tag was generated using complementary oligonucleotides encoding the FLAG sequence (ATG GAC TAC AAA GAC GAT GAC GAT AAA) and BamHI/EcoRI cohesive ends and annealed by mixing equimolar concentrations of each oligonucleotide and incubating at 90°C for 5 min, followed by slow cooling to room temperature. The Myc tag was excised from pCS2-Myc-Dynlt1 using BamHI and EcoRI and the newly synthesized FLAG tag was inserted.

### Yeast two-hybrid assay

Screening of proteins interacting with the C-terminal domain of OX1R was performed in two strains of *Saccharomyces cerevisiae* (Clontech). Clones of AH109 and Y187 strains were tested for their *HIS3* and *β-galactosidase* reporter gene expression, respectively, according to Clontech yeast two-hybrid protocols. Briefly, AH109 yeast cells were successively transformed with pGBKT7-OX1R CTD (fusion with GAL4 DBD), then with a MATCHMAKER mouse brain cDNA library (Clontech) cloned into a yeast GAL4 AD plasmid (pACT2). Successful co-transformations were verified by growth of yeast cells streaked on SD plates lacking L-tryptophan (W) and L-leucine (L) (SD–LW). For the histidine selection test, co-transformed yeast cells were grown on SD–LW plates lacking histidine (H) (SD–LWH), in presence of 5 mM 3-amino-1,2,4-triazole (3-AT), a competitive inhibitor of the product of the HIS3 gene. Colonies were put again on SD–LWH+3-AT plates and plasmids (pACT2) were prepared from the positive colonies. AH109 and Y187 yeast cells were then co-transformed with pGBKT7-OX1R CTD and the pACT2 plasmids to perform *HIS3* and *β-galactosidase* selection assays, respectively. Double-positive plasmids were sequenced.

Subsequent yeast two-hybrid assays were performed by co-transforming pGBKT7-OX1R CTD or pGBKT7-OX2R CTD (wild-type or mutants) with pGADT7-Dynlt1 or pGADT7-Dynlt3 (wild-type or mutants) plasmids in either AH109 or Y187. For the histidine selection test, 3-AT (1 to 25 mM) was added to co-transformed AH109 yeast cells streaked on SD–LWH plates to evaluate strength of interactions. For the β-galactosidase activity assay, co-transformed Y187 yeast cells lysed using freeze/thaw cycles were incubated with ortho-nitrophenyl-β-galactoside. Reactions were stopped with 1 M sodium carbonate when yellow color appeared and absorbance was read at 420 nm. β-galactosidase Miller units were defined as 1000×(OD_420_)/(t×V×OD_600_), where t = time, V = 0.1 mL×concentration factor, OD_600_ = measure of culture cell density.

Protein extracts from AH109 and Y187 co-transformed yeast cells were assessed by western blot using antibodies against tagged fusion proteins [mouse anti-HA (1∶2000, clone HA-7, Sigma, St.Louis, MO) for expression from pGADT7 and rabbit anti-Myc, (1∶1000, ab9106, Abcam, Cambridge, MA) for expression from pGBKT7]. All Dynlt1 fusion proteins were expressed at similar levels in Y187 yeast cells, but expression level of Dynlt1 Δ91–113 was higher than other Dynlt1 constructs in AH109 yeast cells. Dynlt3 was less expressed than mutant forms in Y187 yeast cells, while expression of Dynlt3 Δ1–15 was higher than other Dynlt3 fusion proteins in AH109 (data not shown). All OX1R/OX2R CTD fusion proteins had comparable expression levels in Y187 yeast cells (data not shown).

### Cell culture and transfections

HEK293 cells (ATCC, Manassas, VA, CRL-1573) were maintained in DMEM, supplemented with 10% fetal bovine serum and 2 mM L-glutamine at 37°C with 5% CO_2_. Transient transfections in 24-well culture dishes were done at 70%–80% confluency using Lipofectamine 2000 reagent (Invitrogen, Burlington, Ontario) according to manufacturer recommendations. To generate stable OX1R-GFP cells, HEK293 cells in 6-well plates were transfected with a mixture of OX1R-GFP and a plasmid conferring Neomycin resistance. After 48 hours, cells were diluted and plated in a medium containing G418 (0.8 mg/mL, Invitrogen, Burlington, Ontario). After the death of cells that did not incorporate plasmid DNA (7–10 days), several colonies, each originating from a single cell, were allowed to expand. These colonies were then screened for the expression of GFP-tagged OX1R using a Leica DMIRE2 inverted fluorescent microscope (Leica microsystems, Wetzlar, Germany) to monitor GFP fluorescence. A clone with strong fluorescence in 100% of the cells was used for the experiments, and stability of transfection was ensured by the presence of 0.4 mg/mL G418 in subsequent passages.

### RNA interference

Oligonucleotides targeting human *Dynlt1* and *Dynlt3* genes, along with the negative control siRNA #1, were purchased from Applied Biosystems (Streetsville, Ontario) (references # 139627 and 17984, respectively). HEK293 cells at 60–70% confluency were transfected, 24 hours after seeding, with 0.1 or 0.25 µg of empty pSG5 or pSG5-V5-OX1R in combination with 10 nM or 20 nM siRNA targeted towards *Dynlt1* and/or *Dynlt3* using Lipofectamine 2000 reagent according to manufacturer instructions. Successful inhibition of mRNA expression was verified by quantitative RT-PCR (TaqMan Universal PCR mix) with probes against *Dynlt1* (assay Hs00831821_s1, *Dynlt3* (assay Hs00359622_m1) and *GAPDH* (endogenous control, assay Hs03929097_g1), all from Applied Biosystems. The siRNAs used against *Dynlt1* or *Dyntl3* down-regulated the expression of their target by more than 80%. For experiments combining both siRNAs, reduction of *Dynlt1* and *Dynlt3* expression was 75% and 80%, respectively.

### Immunoprecipitation and immunoblot

Forty-eight hours after transfection, cells were washed twice with PBS and lysed at 4°C in IP buffer [1% NP-40, 150 mM NaCl, 10 mM sodium phosphate, complete mini protease inhibitor (Roche Applied Science, Mannheim, Germany) and 2 µM EDTA]. After removal of insoluble material, 5% of the supernatant was kept at −80°C until immunoblot (IB) analysis. Remaining lysates were mixed with the adequate antibody [mouse anti-V5 (R960, Invitrogen) or mouse anti-GFP (MAB3580, Chemicon, Danvers, MA)] and incubated overnight at 4°C. Protein G-Sepharose beads (Sigma) were washed several times in PBS and were added to the mix for 2 hours at 4°C prior to 4 washes of the beads in IP buffer (without protease inhibitors). Immunoprecipitated proteins were then separated from beads by a 10 min incubation at 95°C and then subjected, together with boiled cell lysates, to SDS–PAGE. Separated proteins were transferred onto nitrocellulose membranes. IB was performed using rabbit anti-Myc antibody (1∶5000, ab9106, Abcam) [or mouse anti-V5 (1∶2000, R960, Invitrogen) or mouse anti-GFP (1∶2500, MAB3580, Chemicon) antibodies to ensure efficient IP]. Subsequently, membranes were incubated with adequate horseradish peroxidase-linked secondary antibody [goat anti-rabbit (1∶10000, 111-035-144, Jackson ImmunoResearch, West Grove, PA) or rabbit anti-mouse (1∶2000, A9044, Sigma)], revealed with enhanced chemiluminescence substrate (Perkin Elmer, Waltham, MA) and exposed to X-ray film.

When proteins extracted from cells transfected with pCS2-Myc-Dynlt1 were subjected to IP with an anti-V5 antibody, IB with anti-Myc antibody consistently yielded a band of the same size as the Myc-tagged Dynlt1 fusion protein. The increase in signal intensity at this height on the films when cells were co-transfected with pCS2-Myc-Dynlt1 and with increasing amounts of pSG5-V5-His-OX1R, but in the presence of a fixed amount of total cDNA, suggests that the band present in the negative controls is due to non-specific binding of Myc-Dynlt1 to the beads during the IP procedure. As for V5-OX1R levels in transfected cells, except for Fig. 5B, we cannot exclude the possibility that the levels of the mutant forms are different from those of the WT receptor. However, this is unlikely, as the OX1R with the whole CTD deletion is expressed at similar levels as the WT receptor (Fig. 5B) and the OX1R (T409A, T412A) mutant shows an initial P-ERK response after OX-A that is very similar to that of WT OX1R (Fig. 6D).

For the assessment of ERK1/2 protein phosphorylation status after stimulation with OX-A, HEK293 cells were transfected with 0.25 µg of empty pSG5 or pSG5-V5-His-OX1R together with 0.125 µg empty pCS2 or pCS2-Myc-Dynlt1. Cells were then exposed to 100 nM OX-A (NeoMPS, Strasbourg, France) from 0 to 60 min. Cell lysates were analyzed by IB, with rabbit anti-phospho-ERK1/2 (1∶2000, #9101, Cell Signaling Technology, Inc., Danvers, MA) and rabbit anti-ERK1/2 (1∶2000, #9102, Cell Signaling Technology, Inc., Danvers, MA) antibodies, followed by incubation with goat anti-rabbit antibody (1∶10000, 111-035-144, Jackson ImmunoResearch). For siRNA experiments, cells were transfected as described in the RNA interference section and stimulated and processed as described in the present paragraph.

### Cell surface ELISA

Enzyme linked immunosorbent assays (ELISA) were performed as described [53]. Briefly, HEK293 cells seeded into poly-L-lysine (0.1 mg/mL) coated wells were transfected with 0.1 or 0.25 µg of empty pSG5 or pSG5-V5-His-OX1R together with 0.125 µg empty pCS2 or pCS2-Myc-Dynlt1. Cells were then stimulated with 100 nM OX-A from 0 to 30 min. On ice, cells were subsequently washed with PBS, fixed in 2% formaldehyde, blocked with 10% FBS, incubated with a rabbit anti-V5 antibody (1∶1000, ab9116, Abcam) followed by incubation with a goat anti-rabbit horseradish peroxidase-linked antibody (1∶1000, 111-035-144, Jackson ImmunoResearch). These complexes were incubated with 200 µL O-phenylenediamine (Thermo Scientific, Rockford, IL) (1 mg/mL) in citrate phosphate buffer (0.5 M each), pH 5.0 for 9 min. Reactions were stopped by addition of an equal volume of 4 N sulfuric acid and then 200 µL was transferred to a 96-well plate for absorbance reading at 490 nm by a microplate reader (Biotek Instruments, 2005). Corrected OD values refer to background-subtracted data at 490 nm (background is the value for HEK293 cells transfected with empty pSG5-V5-His vector). Normalized OD values refer to corrected OD values that have been expressed relative to basal conditions (% of value before stimulation). For siRNA experiments, cells were transfected as described in the RNA interference section and stimulated and processed as described in the present paragraph.

### Immunocytochemistry and co-localization analysis

After transfection with pCS2-Flag-Dynlt1 or empty vector (as described above), stable OX1R-GFP HEK293 cells were stimulated with 100 nM OX-A as above and then fixed with 4% paraformaldehyde. Cells were permeabilized with 0.5% Triton-X100, blocked with 3% bovine serum albumin, followed by incubation with mouse anti-early endosome antigen 1 (EEA1) antibody (1∶500, clone 14/EEA1, BD Biosciences). Alexa Fluor 568 donkey anti-mouse antibody (1∶200, A10037, Invitrogen) was then added, followed by DAPI staining (500 ng/mL) and mounted using Vectashield (Vector Labs, Burlington, Ontario). Negative controls included unlabeled cells (for autofluorescence) as well as cells processed in absence of primary antibody. To verify Dynlt1 transfection efficiency, cells were fixed in methanol, blocked in 3% bovine serum albumin, incubated with mouse anti-FLAG M2 antibody (1∶400, F3165, Sigma) and Alexa Fluor 568 donkey anti-mouse antibody (1∶400, A10037, Invitrogen), followed by DAPI staining. Cells were imaged using a Zeiss Axio Observer.Z1 inverted microscope for transmitted light and epifluorescence with ApoTome attachment (Carl Zeiss, Toronto, Canada). Images obtained using a 40× objective lens were taken with exposure times of 80–100 ms in the red channel (ALEXA568) and 500–700 ms in the green channel (GFP). Images taken in black and white were opened in Openlab Software (Perkin Elmer) and assigned their respective colors. Colored images were then contrast-enhanced in Openlab Software and a region of constant dimension across all images was randomly selected to include 1–3 cells in each image. Images corresponding to OX1R-GFP (green) and endosomes (red) were subsequently co-localized, using the whole image as a region of interest according to guidelines in the Openlab co-localization module (TN348). Thresholds were established in each experiment by using basal conditions (0 min) as the reference for no co-localization. Mander's co-localization co-efficient was generated from the scatterplots of green (OX1R-GFP) and red (EE1A) pixel fluorescence [54], allowing evaluation of the amount of OX1R located in early endosomes.

### Statistical analysis

Data from β-galactosidase assays (yeast two-hybrid) were analyzed by one-way ANOVA followed by Bonferroni correction for multiple comparisons, using Graphpad (GraphPad Software, Inc., La Jolla, CA). The effect of over-expressing or down-regulating Dynlt1 (and Dynlt3) was assessed by two-way ANOVA followed by Tukey's post-hoc test where applicable, using Datasim software (Dr. Bradley, Lewiston, ME). Unpaired Student's *t*-test was performed when comparing the effect of Dynlt1 at a single time point. Statistical significance was set to p<0.05. Data are expressed as means ± SEM.
